# Intramyocardial Injection of siRNAs Can Efficiently Establish Myocardial Tissue-Specific Renalase Knockdown Mouse Model

**DOI:** 10.1155/2016/1267570

**Published:** 2016-10-27

**Authors:** Kun Huang, Ju Liu, Hui Zhang, Jiliang Wang, Huili Li

**Affiliations:** ^1^Institution of Cardiology, Union Hospital, Tongji Medical College, Huazhong University of Science and Technology, Wuhan, Hubei, China; ^2^Department of Geriatrics, Affiliated Hospital of Wuhan Traditional Chinese Medicine and West Medicine, Tongji Medical College, Huazhong University of Science and Technology, Wuhan, Hubei, China; ^3^Department of Art, International Education College, Wuhan University of Technology, Wuhan, Hubei, China; ^4^Department of Gastrointestinal Surgery, Union Hospital, Tongji Medical College, Huazhong University of Science and Technology, Wuhan, Hubei, China

## Abstract

Ischaemia/reperfusion (I/R) injury will cause additional death of cardiomyocytes in ischaemic heart disease. Recent studies revealed that renalase was involved in the I/R injury. So, the myocardial tissue-specific knockdown mouse models were needed for the investigations of renalase. To establish the mouse models, intramyocardial injection of siRNAs targeting renalase was performed in mice. The wild distribution and high transfection efficiency of the siRNAs were approved. And the renalase expression was efficiently suppressed in myocardial tissue. Compared with the high cost, time consumption, and genetic compensation risk of the Cre/loxP technology, RNA interference (RNAi) technology is much cheaper and less time-consuming. Among the RNAi technologies, injection of siRNAs is safer than virus. And considering the properties of the I/R injury mouse models, the efficiency and durability of injection with siRNAs are acceptable for the studies. Altogether, intramyocardial injection of siRNAs targeting renalase is an economical, safe, and efficient method to establish myocardial tissue-specific renalase knockdown mouse models.

## 1. Introduction

Ischaemic heart disease is one of the most common coronary artery diseases [1–3]. Restoration of blood flow can improve the clinical outcomes [4, 5], but reperfusion after ischaemia will cause additional death of cardiomyocytes in a process known as I/R injury. Recent evidence has implicated the potential protective roles of renalase in cardiomyocytes in the process of I/R injury [6–10]. To investigate the function of renalase, the technology of gene knockdown or knockout will be used. Conventional gene knockdown or knockout animal models are systemic and non-organ-specific. It will always cause some unpredictable abnormalities in other organs beside the target ones, which will influence the phenotypes of the animal models. Instead, the animal models with organ-specific gene knockdown or knockout will be a better choice. Therefore, to investigate the roles of renalase in cardiomyocytes in the process of I/R injury* in vivo*, specifically knockdown or knockout of renalase in myocardial tissue of mouse models will be the most effective method. Both the Cre/loxP technology and the RNAi technology can be used to establish the organ-specific knockdown or knockout mouse models. Intramyocardial injection of siRNAs targeting renalase is the most economical method, which is affordable for most researchers. But the efficiency has not been sufficiently demonstrated. In the present study, the operating method and knockdown efficiency of the intramyocardial injection of siRNAs targeting renalase were explored and verified in mouse models. We demonstrated that intramyocardial injection of siRNAs targeting renalase was an efficient and economical way to generate the myocardial tissue-specific renalase knockdown mouse models. This model provides a proper base for the researches of renalase in the process of myocardial I/R injury.

## 2. Materials and Methods

### 2.1. Ethics Statement

All animal experiments were performed in accordance with the National Institutes of Health (NIH) Guide for the Care and Use of Laboratory Animals published by the US National Institutes of Health (NIH Publication, 8th edition, 2011) and approved by the Ethics Committee of Tongji Medical College, the Huazhong University of Science and Technology, China.

### 2.2. RNA Interference

Cy3-labeled cholesterol-conjugated-specific siRNAs for renalase (sense: 5′-GUGGCACCUCAAGGAAUUUCUdTdT-3′; antisense: 3′-dTdT CACCGUGGAGUUCCUUAAAGA-5′) were purchased from Ribobio Co., Guangzhou, China. RNLS (GTGGCACCTCAAGGAATTTCT) was chosen as target region.

### 2.3. *In Vivo* Delivery of siRNAs

**Figure 1:**
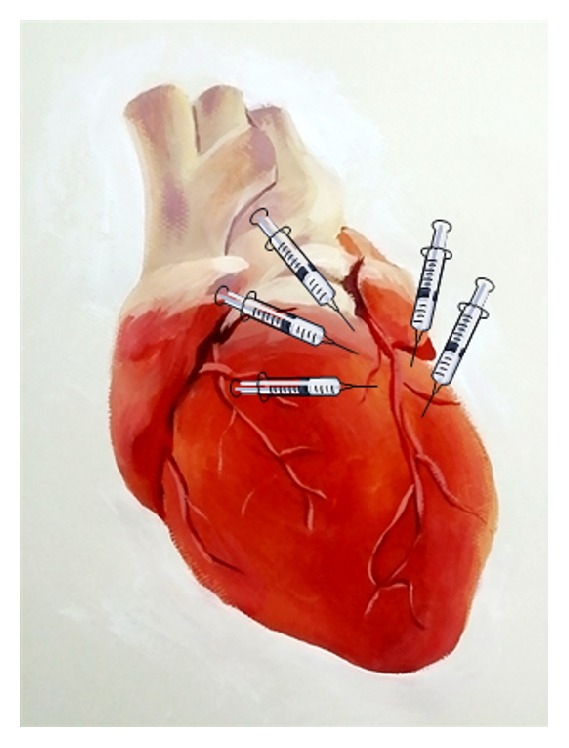
Intramyocardial injection into the LV in approximately five sites surrounding the initiation part of the LAD coronary artery. The injectors indicate the injection sites.

*In vivo* siRNAs delivery was performed as previously described [10–12]. Male C57BL/6 mice, aged 6–8 weeks, were purchased from Beijing University (Beijing, China) and maintained on a chow diet in a 12 h light/12 h dark environment at 25°C at the Tongji Medical College Animal Care Facility. Mice were anaesthetized with pentobarbital sodium (50 mg/kg) by an intraperitoneal injection, orally intubated, and connected to a rodent ventilator. Different concentrations (0 mM, 1 mM, 2 mM, and 3 mM) of 20 *μ*L Cy3-labeled cholesterol-conjugated-specific siRNAs for renalase (Ribobio Co., Guangzhou, China) were intramyocardially injected with a 32 G needle into the left ventricle (LV) in approximately five sites surrounding the initiation part of the left anterior descending (LAD) coronary artery (Figure 1). The siRNAs solutions were prepared according to the manufacturer's instructions. At the time point of 12 h after the siRNAs injection, mice were euthanized with pentobarbital sodium (200 mg/kg) by an intraperitoneal injection, and myocardial tissue of the LV was isolated and used for further assays. Furthermore, at different time points (0 h, 6 h, 12 h, and 24 h) after the siRNAs injection (3 mM, 20 *μ*L), mice were euthanized and myocardial tissue of the LV was isolated and used for further assays. Frozen sections and fluorescent microscope imaging were performed to detect the distributions and transfection efficiencies of siRNAs. Western blotting and real-time qPCR were performed to detect the protein levels and mRNA expressions of renalase in myocardial tissue of the LV.

### 2.4. *In Vivo* Myocardial I/R Model

Surgical induction of myocardial I/R was performed as previously described [10, 13, 14]. Briefly, mice were anaesthetized by an intraperitoneal injection with pentobarbital sodium (50 mg/kg), orally intubated, connected to a rodent ventilator, and placed in the supine position. A left thoracotomy was performed. Left anterior descending (LAD) coronary artery was visualized and ligated using a 6-0 silk suture around fine PE-10 tubing with a slip knot. Mice were subjected to 30 min of LAD ischaemia followed by varying periods of reperfusion, respectively. In the sham group, a suture was passed under the LAD but not tied. After experiments, mice were euthanized with pentobarbital sodium (200 mg/kg) by an intraperitoneal injection, and the ischaemic-reperfused tissue was isolated and used for histological analysis.

### 2.5. Histology

The myocardial tissues were subjected to frozen sections. Fluorescent microscope imaging was used for the detection and characterization of the distributions and transfection efficiencies of the Cy3-labeled cholesterol-conjugated siRNAs. Fluorescence was detected at excitation and emission wavelengths of 554 nm and 574 nm, respectively. The tissue sections were imaged under a fluorescent microscope (IX71, Olympus). The density of fluorescent was analyzed by Image J 1.44p software.

### 2.6. Western Blot and Antibodies

As described in previous studies [15, 16], total tissues were lysed using RIPA buffer, and the protein concentration was determined with a BCA protein assay kit (Pierce Company, Rockford, IL, USA). Protein extracts were used for SDS-PAGE (Invitrogen, Carlsbad, CA, USA), and the proteins were transferred to a polyvinylidene fluoride membrane (Millipore, Billerica, MA, USA), which was incubated with primary antibodies (renalase GTX89570, GeneTex, diluted 1 : 1000) overnight at 4°C. After incubation with secondary antibodies (goat IgG antibody GTX228416-01, GeneTex, diluted 1 : 5000) for 1 h at room temperature, the membranes were treated with ECL reagents (170-5061, Bio-Rad, Hercules, CA, USA) prior to visualization using a FluorChem E imager (Cell Biosciences, San Jose, CA) according to the manufacturer's instructions. The specific protein expression levels were normalized to *β*-tubulin on the same nitrocellulose membrane.

### 2.7. RNA Extraction and Real-Time qPCR

As previously described [2, 17], total RNA was isolated using Trizol reagent (Invitrogen, Carlsbad, CA, USA) according to the manufacturer's instructions. 2 *μ*g of total RNA was reversely transcribed using RNA PCR Kit (Takara Biotechnology, Otsu, Japan), and the resulting cDNA was used as a PCR template. The mRNA levels were determined by real-time qPCR with an ABI PRISM 7900 Sequence Detector system (Applied Biosystem, Foster City, CA, USA) according to the manufacturer's instructions and normalized to *β*-actin gene expression. The experiment was performed in triplicate. The sequences of primers for real-time qPCR are listed in Table 1.

### 2.8. Statistical Analysis

As described in previous studies [18–20], the statistical analysis was performed with the Statistical Package for Social Sciences (version 13.0; SPSS, Chicago, IL). All the data were expressed as mean ± SD (standard deviation, SD) and the difference was analyzed by one-way ANOVA. Statistical analysis was performed using Student's *t*-test for paired data. The difference was considered as statistically significant when *p* value was less than 0.05.

## 3. Results

### 3.1. The Cy3-Labeled Cholesterol-Conjugated-Specific siRNAs Can Spread Wildly into the Myocardial Tissue of the LV by the Method of Intramyocardial Injection

**Figure 2:**
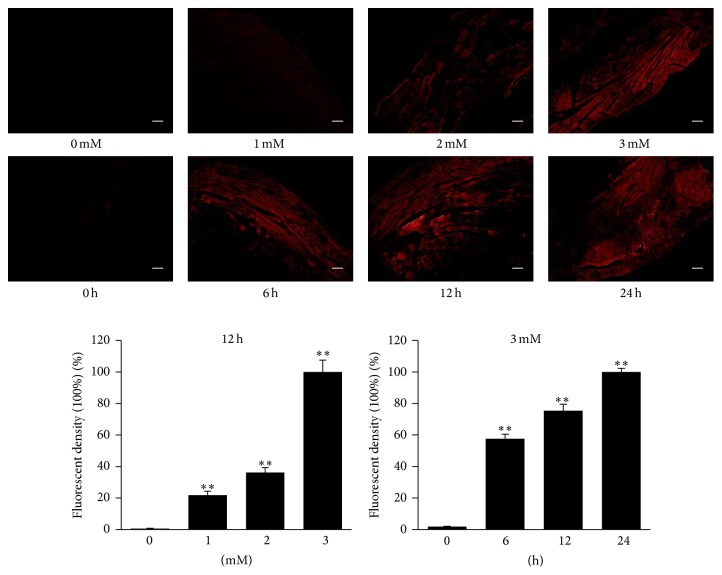
The fluorescent density increased in a time-dependent and dose-dependent manner. Myocardial tissues at 12 h after intramyocardial injection of different concentrations (0 mM, 1 mM, 2 mM, and 3 mM) of 20 *μ*L Cy3-labeled cholesterol-conjugated-specific siRNAs and at different time points (0 h, 6 h, 12 h, and 24 h) after the siRNAs injection (3 mM, 20 *μ*L) were subjected to frozen section and fluorescent imaging. The fluorescent density of each group was analyzed. Scale bar represents 200 *μ*m. *∗∗* indicates significant differences; *p* < 0.01. Data are plotted as the mean ± SD from five independent experiments. Bars indicate the standard deviation of the mean.

We isolated the myocardial tissue which had been injected with the Cy3-labeled cholesterol-conjugated-specific siRNAs for renalase. Frozen sections and fluorescent microscope imaging were performed. We found that the red fluorescence of Cy3 is distributed wildly in the myocardial tissue of the LV (Figure 2). The fluorescent density increased after the siRNAs injection in a dose-dependent and time-dependent manner. As shown in Figure 2, under the condition of the same injection volume (20 *μ*L), the fluorescent density increased significantly with the increase of the siRNAs concentration (*p* < 0.01; Figure 2). Meanwhile, under the same concentration and injection volume, at different time points, the fluorescent density increased significantly with time (*p* < 0.01; Figure 2).

### 3.2. Intramyocardial Injection of the Cy3-Labeled Cholesterol-Conjugated-Specific siRNAs Targeting Renalase Can Efficiently Knock Down Renalase in Myocardial Tissue

**Figure 3:**
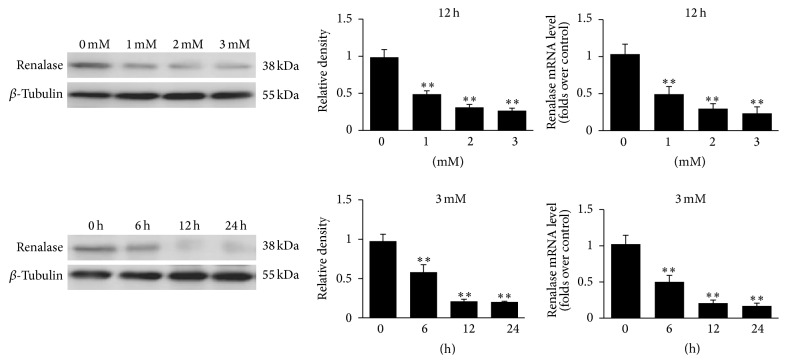
Renalase protein levels and expressions were suppressed by the siRNAs in a time-dependent and dose-dependent manner. Western blot analysis and real-time qPCR of renalase in myocardial tissues at 12 h after intramyocardial injection of different concentrations (0 mM, 1 mM, 2 mM, and 3 mM) of 20 *μ*L Cy3-labeled cholesterol-conjugated-specific siRNAs and at different time points (0 h, 6 h, 12 h, and 24 h) after the siRNAs injection (3 mM, 20 *μ*L). Densitometric analysis of Western blot of renalase was carried out. *∗∗* indicates significant differences; *p* < 0.01. Data are plotted as the mean ± SD from five independent experiments. Bars indicate the standard deviation of the mean.

We isolated the myocardial tissue which had been injected with the Cy3-labeled cholesterol-conjugated-specific siRNAs for renalase. Myocardial tissue samples were examined by Western blot analysis and real-time quantitative PCR (real-time qPCR). Overall, these results revealed that renalase protein levels (*p* < 0.01; Figure 3) and mRNA expressions (*p* < 0.01; Figure 3) were significantly attenuated in myocardial tissue samples in a dose-dependent and time-dependent manner. Under the condition of the same injection volume (20 *μ*L), the protein levels and mRNA expressions of renalase decreased significantly with the increase of the siRNAs concentration (*p* < 0.01; Figure 3). Meanwhile, under the same concentration and injection volume, at different time points, the protein levels and mRNA expressions of renalase decreased significantly with time (*p* < 0.01; Figure 3).

### 3.3. The Spared Area and Transfection Efficiencies of the Cy3-Labeled Cholesterol-Conjugated-Specific siRNAs Targeting Renalase Are the Same between the Sham and I/R Operation Groups

**Figure 4:**
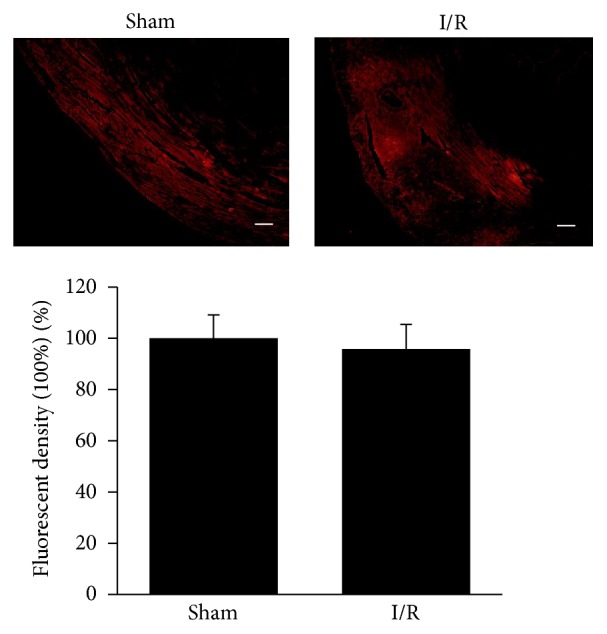
There is no difference of fluorescent density between the sham and I/R operation groups. 24 h after intramyocardial injection of Cy3-labeled cholesterol-conjugated-specific siRNAs (3 mM, 20 *μ*L), sham and I/R operations were performed; myocardial tissues were subjected to frozen section and fluorescent imaging. The fluorescent density of each group was analyzed. Scale bar represents 200 *μ*m. Data are plotted as the mean ± SD from five independent experiments. Bars indicate the standard deviation of the mean.

To compare the spared area and transfection efficiencies of the Cy3-labeled cholesterol-conjugated-specific siRNAs targeting renalase between the sham and I/R operation groups,* in vivo* myocardial I/R models were performed 24 h after intramyocardial injection of the Cy3-labeled cholesterol-conjugated-specific siRNAs (3 mM, 20 *μ*L) for renalase. Myocardial tissue was isolated; frozen sections and fluorescent microscope imaging were performed. We found that the red fluorescence of Cy3 is distributed wildly in the myocardial tissue of the LV in both the sham and I/R operation groups (Figure 4). There is no significant difference of fluorescent density between the sham and I/R operation groups (*p* > 0.05; Figure 4). The renalase expression of both the sham and I/R operation groups was efficiently suppressed by the siRNAs (data not shown).

## 4. Discussion

Increasing evidence has implicated the potential roles of renalase in ischaemic heart disease. Renalase knockdown or knockout animal models, especially organ-specific or tissue-specific knockdown or knockout, were urgently needed. In the current study, we focused on the operating method and knockdown efficiency of intramyocardial injection of siRNAs targeting renalase. Attenuated myocardial renalase levels and enhanced Cy3-fluorescent density in myocardial tissues were found in the mouse models. The cholesterol-conjugated-specific siRNAs can spread wildly into the myocardial tissue. And the transfection efficiency and knockdown efficiency of renalase by siRNAs* in vivo* are high enough to suppress the expression of renalase. Our findings suggest that intramyocardial injection of siRNAs targeting renalase is an efficient method to generate myocardial tissue-specific renalase knockdown mouse models. It provides a proper base for the researches of renalase in the myocardial I/R injury mouse models. And its efficiency and feasibility have been proved after myocardial I/R operation. Theoretically, this method is a better choice, compared with the Cre/loxP technology and intramyocardial injection of virus, to establish a basic mouse model (renalase knockdown) for I/R operation.

Cre/loxP technology [21] has allowed the generation of organ-selective, tissue-selective, or cell-selective knockout models targeting a specific gene [22]. The site-specific recombinase Cre (cyclization recombination) from the bacteriophage P1 was used in this technology. The Cre is used to induce recombination between two recognition sites (termed loxP-sites, locus of crossing [X-ing] over in P1) inserted in the genome. Two inverted repeat sequences surrounding a core sequence are contained in loxP-sites, whose directionality was given by the sequences. LoxP-sites are often described as arrowheads in genomic sequences because of their directionality. Two transgenic mouse strains are needed to generate an organ or tissue-selective knockout: one strain in which the Cre recombinase is expressed under the control of a suitable organ or tissue-selective promoter and a second strain in which the target gene, or a functionally critical exon, is flanked by two loxP-sites (“floxed”). After one round of crossbreeding, double transgenic progeny carrying both the floxed transgene and the Cre transgene can be selected. In the second round of breeding, these double transgenic mice are either inbred or crossbred with the original floxed mice. In the resulting F2 generation, pups that are homozygous for the floxed allele and that carry the Cre transgene (either in homozygous or in heterozygous state) are selected. In the latter animals, the floxed gene/exon will be excised selectively in the cell types that express Cre recombinase [22]. Therefore, establishing animal models using Cre/loxP technology is extremely expensive and time-consuming. Recently, it was reported that deleterious mutations, known as gene knockouts, would induce genetic compensation [23]. Genetic compensation is a mechanism of cells to adapt their environment by fine-tuning their transcriptome. It is gene expression control to compensate for gene dosage in cells. Once a gene was deleteriously mutated or knocked out by other technics, other related genes will be upregulated to compensate or replace the role of the mutated gene in cells. Genetic compensation will cause invalid gene knockout, which results in animal models without phenotypes. But gene knockdowns would not induce the genetic compensation mechanism. Thus, using gene deleterious mutation or Cre/loxP technology to generate a gene knockout animal model has the risk of inducing the genetic compensation mechanism, which may lead to invalid gene knockout. Additionally, RNAi technology is much cheaper and less time-consuming, and its efficiency is acceptable in most situations. Therefore, using RNAi technology to generate organ or tissue-specific gene knockdown models may be a better choice compared with the knockout or Cre/loxP technology.

Selectively knockdown of renalase in myocardial tissue can be achieved by intramyocardial injection of either siRNAs targeting renalase or virus (adenovirus, lentivirus, or adenoassociated virus which contains the plasmid of shRNAs targeting renalase). Intramyocardial injection of virus is usually used in myocardial infarction models or other long-term investigations. It aims to establish a myocardial tissue-specific knockdown mouse model which can stably express shRNAs targeting the gene of interest [24–26]. Once the virus is injected and the mouse models are established, the expression of the target gene can be stably and persistently suppressed. Instead, injection of siRNAs can establish a tissue-specific knockdown mouse model rapidly [12, 27, 28]. Correspondingly, the suppressive effect to the expression of the target gene will not last for a long time, just several days or weeks. And the knockdown efficiency will attenuate with time. In our studies, it just needs several hours to establish the* in vivo* myocardial I/R models [10, 13, 14]. Thus, it is unnecessary to using virus to establish the renalase stably knockdown mouse models. Furthermore, the safety of virus using either to mouse or to researchers needs to be considered. It has been reported that local myocardial inflammation and fibrosis in the LV were proportional to transduction efficiency in intramyocardial injection of virus [24]. Additionally, operating with virus, researchers will be at risk of infection. Therefore, intramyocardial injection of siRNAs targeting renalase will be an economical, a little time-consuming, and relatively safe method to establish myocardial tissue-specific renalase knockdown mouse models. And the knockdown efficiency and durability are acceptable.

In conclusion, our study showed that intramyocardial injection of siRNAs targeting renalase could successfully establish myocardial tissue-specific renalase knockdown mouse models. This method is economical and efficient. The required materials are accessible and affordable for most researchers. Furthermore, the knockdown efficiency of renalase is high enough to effectively suppress the expression of renalase. Therefore, it is a suitable myocardial tissue-specific renalase knockdown mouse model for the researches of the myocardial I/R injury.

## Figures and Tables

**Table 1:** The sequences of primers for real-time qPCR.

Name	Use	Orientation	Sequence
Renalase	Real-time qPCR	F	5′-AGTGAACGCCAGAGGGAGCAA-3′
		R	5′-TAGCGGCAGGACCAAGGGAC-3′
*β*-Actin	Real-time qPCR	F	5′-AACAGTCCGCCTAGAAGCAC-3′
		R	5′-CGTTGACATCCGTAAAGACC-3′
